# Exposure to the antiretroviral drug dolutegravir impairs structure and neurogenesis in a forebrain organoid model of human embryonic cortical development

**DOI:** 10.3389/fnmol.2024.1459877

**Published:** 2024-11-06

**Authors:** Emma LaNoce, Daniel Y. Zhang, Alan Garcia-Epelboim, Yijing Su, Yusha Sun, Giana Alepa, Angelina R. Angelucci, Cagla Akay-Espinoza, Kelly L. Jordan-Sciutto, Hongjun Song, Guo-li Ming, Kimberly M. Christian

**Affiliations:** ^1^Department of Neuroscience and Mahoney Institute for Neurosciences, Perelman School of Medicine, University of Pennsylvania, Philadelphia, PA, United States; ^2^Department of Neurosurgery, Perelman School of Medicine, University of Pennsylvania, Philadelphia, PA, United States; ^3^Department of Oral Medicine, School of Dental Medicine, University of Pennsylvania, Philadelphia, PA, United States; ^4^Neuroscience Graduate Group, Perelman School of Medicine, University of Pennsylvania, Philadelphia, PA, United States; ^5^Institute for Regenerative Medicine, University of Pennsylvania, Philadelphia, PA, United States; ^6^Department of Cell and Developmental Biology, Perelman School of Medicine, University of Pennsylvania, Philadelphia, PA, United States; ^7^The Epigenetics Institute, Perelman School of Medicine, University of Pennsylvania, Philadelphia, PA, United States; ^8^Department of Psychiatry, Perelman School of Medicine, University of Pennsylvania, Philadelphia, PA, United States

**Keywords:** antiretrovirals, brain organoids, iPSCs, neurodevelopment, dolutegravir, HIV-1

## Abstract

**Introduction:**

For many therapeutic drugs, including antiretroviral drugs used to treat people living with HIV-1 (PLWH), we have little data on the potential effects on the developing human brain due to limited access to tissue and historical constraints on the inclusion of pregnant populations in clinical trials. Human induced pluripotent stem cells (iPSCs) offer a new avenue to gain insight on how drugs may impact human cell types representative of the developing central nervous system. To prevent vertical transmission of HIV and promote the health of pregnant PLWH, antiretroviral therapy must be initiated and/or maintained throughout pregnancy. However, many antiretroviral drugs are approved for widespread use following clinical testing only in non-pregnant populations and there may be limited information on potential teratogenicity until pregnancy outcomes are evaluated. The integrase strand transfer inhibitor dolutegravir (DTG) is a frontline antiretroviral drug that is effective in viral suppression of HIV but was previously reported to be associated with a slight increase in the risk for neural tube defects in one study, although this has not been replicated in other cohorts.

**Methods:**

To directly investigate the potential impact of DTG on human cortical neurogenesis, we measured the effects of daily drug exposure on the early stages of corticogenesis in a human iPSC-based forebrain organoid model. We quantified organoid size and structure and analyzed gene and protein expression to evaluate the impact of several doses of DTG on organoid development.

**Results:**

We observed deficits in organoid structure and impaired neurogenesis in DTG-treated organoids compared to vehicle-treated control organoids after 20 or 40 days in culture. Our highest dose of DTG (10 μM) resulted in significantly smaller organoids with a reduced density of neural rosette structures compared to vehicle-treated controls. Mechanistically, RNA-sequencing and immunohistological analysis suggests dysregulated amino acid transport and activation of the integrated stress response in the DTG-treated organoids, and functionally, a small molecule integrated stress response inhibitor (ISRIB) could partially rescue increased expression of proteins related to cell cycle regulation.

**Discussion:**

Together, these results illustrate the potential for human iPSC-based strategies to reveal biological processes during neurogenesis that may be affected by therapeutic drugs and provide complementary data in relevant human cell types to augment preclinical investigations of drug safety during pregnancy.

## 1 Introduction

Antiretroviral therapy (ART) has been remarkably successful in diminishing viral load and improving quality of life for people living with HIV-1 (PLWH) (Bekker et al., 2023). ART typically consists of two or more antiretroviral drugs from different mechanistic classes that include inhibitors of integrase, protease, reverse transcriptase, fusion, and receptor binding and entry (Menendez-Arias and Delgado, 2022). Given that antiretroviral drugs are so highly effective in suppressing viral activity, pregnant populations and individuals trying to conceive are advised to initiate or continue ART to prevent vertical transmission, even when weighed against the unknown potential teratogenicity of several ART drugs (United States Department of Health and Human Services, 2024). As new drugs and formulations are developed to streamline multi-agent regimens and improve adherence, the safety of these drugs for use by pregnant PLWH remain an ongoing concern (Eke et al., 2023; Gibas et al., 2022). Historically, pregnant populations have often been excluded from clinical trials and there are limited data on the impact of many therapeutic drugs on human fetal brain development (Schnoll et al., 2021; Shields and Lyerly, 2013). Much of our current data is obtained from observational studies of pregnancy outcomes following the approval of drugs tested in the general population (Wang et al., 2019). For antiretroviral drugs, several observational studies have been conducted or are ongoing, focusing on birth outcomes, with some studies extending analysis to early development, which have provided critical data on rates of vertical transmission, maternal health, and neurodevelopmental effects of various ART regimens during pregnancy (Boivin et al., 2019; Dadabhai et al., 2019; Gibb et al., 2012; Malaba et al., 2017). These studies have contributed to the evolving guidelines from government agencies regarding several antiretroviral drugs including zidovudine and efavirenz (Schnoll et al., 2021) and remain a critical pipeline for the ongoing evaluation of drug safety and efficacy.

In advance of a recommendation for expanded use of the integrase strand transfer inhibitor (INSTI) dolutegravir (DTG) by the World Health Organization Guideline Development Group, an interim analysis of an ongoing observational study in Botswana reported 4 instances of neural tube defects among 426 infants (0.94%) born to women who had been taking DTG at the time of conception compared to 14 instances among 11,300 infants (0.12%) born to women taking other ART drugs, with a difference in prevalence calculated to be −0.82% (Zash et al., 2018). International health agencies issued statements of concern that included a Drug Safety Communication from the U.S. Food and Drug Administration in 2018 and a temporary reclassification of DTG from a preferred to an alternative drug by the U.S. Department of Health and Human Services (Mofenson et al., 2019). Subsequent studies showing little to no effect of DTG on the occurrence of neural tube defects have led to a consensus that the risk is minimal and is outweighed by the general tolerability and efficacy of DTG (Eke et al., 2023; Kourtis et al., 2023; Mofenson et al., 2019; Patel et al., 2022; Pereira et al., 2021; Zash et al., 2019). However, there remain open questions as to whether DTG has any direct impact on human neural progenitors that are representative of the early developing nervous system and, if so, which biological processes and pathways are affected.

Human induced pluripotent stem cells (iPSCs) can be generated by reprogramming adult somatic cells and provide a versatile resource to generate most cell types, including those of the central and peripheral nervous systems within a human genetic context (Takahashi et al., 2007). These iPSCs offer new opportunities for disease modeling (Eichmuller and Knoblich, 2022) and to evaluate the impact of drugs (Silva and Haggarty, 2020), environmental toxins (Fan et al., 2022), genetic risk factors (Bicks and Geschwind, 2024), and viruses (Su et al., 2021) on human neural cell types, which would otherwise be inaccessible. Targeted differentiation of iPSCs has revealed cell type-specific responses to various perturbagens in highly enriched populations of human neural cells (Shi et al., 2017; Tao and Zhang, 2016). Human iPSCs can also be cultured under conditions that allow for the development of organoids, 3D structures that self-organize to model several tissue-level features of the developing brain and allow for the investigation of higher order properties such as structural defects reminiscent of cephalic disorders (Fernando et al., 2020; Kanton et al., 2019; Velasco et al., 2019; Zhang et al., 2021). iPSC cultures start from an undifferentiated stem cell state, and most brain organoid models are well-aligned with early neurodevelopment based on gene expression, cell composition, and functional properties (Qian et al., 2019). Recent studies have begun to use organoids to model the impact of HIV and antiretroviral drugs on the central nervous system (Donadoni et al., 2024; Premeaux et al., 2021). Many of these studies have focused on generating organoids with integrated microglia to allow for the investigation of productive infection of HIV and its effect on neural cell types (Gumbs et al., 2022; Kong et al., 2024). Only a few have focused on the direct impact of antiretroviral drugs, including DTG, and shown its impact on morphogenesis, organoid structure, and expression of the folate receptor alpha (Caiaffa et al., 2024; Kirkwood-Johnson et al., 2021). We have optimized a forebrain cortical organoid protocol that captures several features of cortical development, including the emergence of rosette structures with polarized progenitor zones (Qian et al., 2018). This organoid model has been used to identify Zika virus-mediated effects on neural progenitors and structural organization that resemble some features of congenital Zika syndrome in infants (Qian et al., 2016) and can be an effective platform to investigate the developmental impact of therapeutic drugs on human neural cell types.

Motivated by the early signal of increased risk for neural tube defects (Zash et al., 2019; Zash et al., 2018) and recent reports showing neurodevelopmental impairments in mice following DTG exposure (Bade et al., 2021; Mohan et al., 2021; Tukeman et al., 2024), we evaluated the impact of DTG, as well as another INSTI, raltegravir (RTG), on early-stage cortical organoids derived from human iPSCs. We used several doses of DTG (0.1–10 μM) within the range of cord blood concentrations reported in the literature (Lewis et al., 2016; Mulligan et al., 2018; Pain et al., 2015; Rimawi et al., 2017; Schalkwijk et al., 2016). Our results suggest that sustained exposure to 10 μM DTG, but not 10 μM RTG, impacts organoid structure and organization and leads to a deficit in neurogenesis. RNA sequencing revealed a downregulation of genes related to neurogenesis and an upregulation of stress-associated genes and suggests the involvement of the integrated stress response (ISR). This study provides a proof-of-principle to demonstrate the utility of iPSC-based models to generate complementary data that can be used to evaluate the impact of drugs on neural stem cells and the developing human nervous system.

## 2 Materials and methods

### 2.1 Human induced pluripotent stem cell lines

Human iPSC lines used in the current study were either obtained from commercial sources or previously generated and fully characterized (Chiang et al., 2011; Wen et al., 2014) (Supplementary Table 1). C1-2 iPSCs were generated from fibroblasts obtained from ATCC (CRL-2097). C3-1 iPSCs were generated from fibroblasts obtained from an individual in a previously characterized American family (Sachs et al., 2005). WTC11 iPSCs were obtained from Coriell Institute for Medical Research (GM25256). All lines were derived from skin fibroblasts that were reprogrammed using an integration-free episomal vector approach and characterized through karyotyping, expression of markers for pluripotent stem cells such as Nanog, Oct4, and SSEA4, and the capacity to generate cell types representative of all three germ layers of embryonic development (Chiang et al., 2011; Kreitzer et al., 2013; Okita et al., 2011; Wen et al., 2014; Yu et al., 2009). Generation of the C1-2 and C3-1 iPSC lines followed institutional IRB and ISCRO guidelines and was approved by the Johns Hopkins University School of Medicine. Karyotyping analysis by standard G-banding technique was carried out by the Cytogenetics Core Facility at the Johns Hopkins Hospital or Cell Line Genetics. Results were interpreted by clinical laboratory specialists of the Cytogenetics Core or Cell Line Genetics. All iPSC lines were confirmed free of Mycoplasma, Acholeplasma, and Spiroplasma with a detection limit of 10 CFU/mL by targeted PCR (Biological Industries).

### 2.2 Human induced pluripotent stem cell culture

All studies involving human iPSCs were performed under approved protocols of the University of Pennsylvania. For cortical organoid generation, iPSCs were cultured on mouse embryonic fibroblast feeder (MEF) cells in stem cell medium consisting of Dulbecco’s Modified Eagle Medium/Nutrient Mixture F-12 (DMEM:F12) supplemented with 20% KnockOut Serum Replacement, 1X MEM-non-essential amino acids (NEAA), 1X GlutaMAX, 1X penicillin-streptomycin, 1X 2-mercaptoethanol, and 10 ng/mL bFGF in a 5% CO_2_, 37°C, 90% relative humidity incubator as previously described with minor modifications (Qian et al., 2018) (see Supplementary Table 1 for catalog numbers for all reagents). Heat-stable bFGF (Gibco) was used at the same concentration to replace the previously reported recombinant bFGF in the iPSC culture. Culture medium was replaced every day. iPSCs were passaged every week onto a new tissue-treated culture plate coated with 0.1% gelatin for more than 4 h and pre-seeded with γ-irradiated CF1 MEF cells one day in advance. Colonies were detached by washing with DPBS and treated with 1 mg/mL collagenase type IV for 30–60 min. Detached colonies were washed 3 times with 5 mL DMEM:F12 and dissociated into small clusters by trituration with a P1000 pipette.

### 2.3 Organoid culture and drug treatments

**FIGURE 1:**
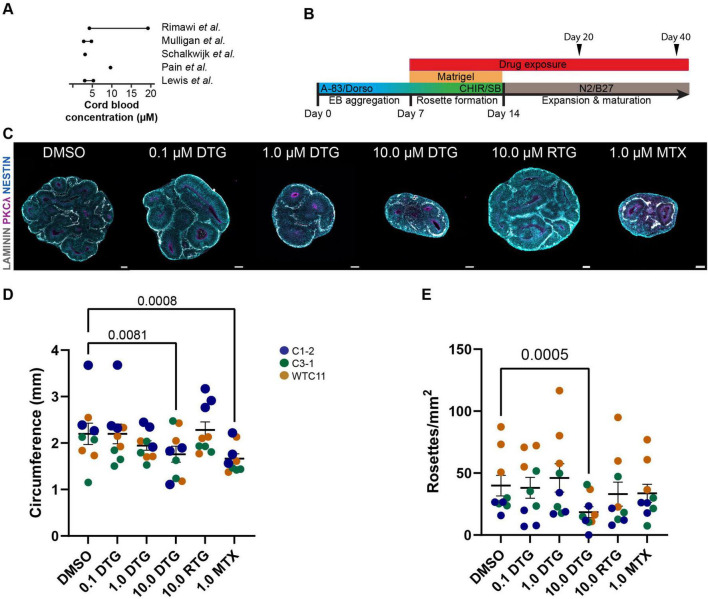
High dose DTG decreases organoid size and number of rosettes. **(A)** Summary schematic showing reported concentrations of DTG in umbilical cord blood ranging from ∼3 to 20 μM, which was used to determine the range of DTG used in the present study (0.1, 1.0, or 10.0 μM). **(B)** Schematic of the experimental protocol showing timeline of days in vitro with administration of DTG, RTG, MTX, or DMSO starting at Day 7 and continuing with daily media changes until the samples were collected for processing at Day 20 or Day 40. Embryoid bodies (EB) were embedded in Matrigel at Day 7 at the initiation of drug administration and extracted on Day 14 for further differentiation of organoids on an orbital shaker through the remainder of the experiment. A-83: A 83-01 and SB: SB-431542, inhibitors of TGFβ type 1 receptors ALK5, ALK4 and ALK7; Dorso: dorsomorphin, inhibitor of bond morphogenic protein; CHIR: CHIR99021, β-catenin activator; N2, B27, to promote neural differentiation and survival of neurons. **(C)** Representative images showing the impact of DTG, RTG, and MTX on organoid size compared to DMSO. Scale bars, 100 μm. **(D,E)** Quantification of the circumference **(D)** and number of rosettes **(E)** of organoids from 3 iPSC lines with 3 independent experiments for each line. Each marker represents the circumference or number of rosettes averaged over 3 organoids for each experimental batch and condition. Marker color denotes the identity of the iPSC line. Two-way ANOVA revealed a significant effect of cell line identity as well as drug treatment on both organoid circumference and density of rosettes (circumference: cell line (F_8,148_ = 8.636, *p* < 0.0001) and drug treatment (F_5,148_ = 6.864, *p* < 0.0001); rosette density: cell line (F_8,148_ = 20.44, *p* < 0.0001) and drug treatment (F_5,148_ = 6.091, *p* < 0.0001). Post hoc analyses with Dunnett’s test for multiple comparisons show an effect of drug exposure for 10.0 μM DTG and 1.0 μM of MTX on circumference and 10.0 μM DTG on rosette density compared to DMSO-treated organoids. Adjusted *p*-values are shown for statistically significant differences among pairwise comparisons. Data shown are means ± SEM.

Forebrain organoids were cultured from iPSCs as previously described (Qian et al., 2018; Qian et al., 2016), with some modifications. Briefly, hiPSC colonies were detached with collagenase type IV, washed with DMEM:F12, and transferred to an uncoated polystyrene petri dish (VWR) in neural induction medium containing DMEM:F12 supplemented with 20% KnockOut Serum Replacement, 1X MEM-NEAAs, 2mM GlutaMax, penicillin (100 units/mL)/streptomycin (100 μg/mL), 0.1mM 2-mercaptoethanol, 2 mM dorsomorphin and 2 mM A83-01. On Day 5 and Day 6, half of the medium was replaced with forebrain induction medium consisting of DMEM:F12 supplemented with 1X N2 supplement, penicillin (100 units/mL)/streptomycin (100 μg/mL), 1X NEAA, 2mM GlutaMax, 1 mM CHIR99021, and 1 mM SB-431542. On Day 7, organoids were embedded in Matrigel and cultured in forebrain induction medium with drug or vehicle supplementation for 7 more days. On Day 14, embedded organoids were dissociated from Matrigel by washing 3 times with 2 mL ice-cold Dulbecco’s Phosphate-Buffered Saline (DPBS, Gibco), incubated in 4 mL Cell Recovery Solution (Corning) at 4°C with gentle rotation or rocking, and finally washed 3 times with 2 mL ice-cold DPBS. Incubation time in cell recovery solution ranged from 15 to 30 min with periodic visual inspection to determine the optimal time for maximal Matrigel dissolution while maintaining good organoid integrity. Organoids were transferred to a 6 well-plate on a CO_2_ resistant orbital shaker (Thermo Scientific) and grown in differentiation medium consisting of DMEM:F12 supplemented with 1X N2 supplement, 1X B27 supplement, 1X penicillin (100 units/mL)/streptomycin (100 μg/mL), 0.1mM 2-mercaptoethanol, 1X NEAA, and 2.5 g/mL human insulin. Stocks of DTG (Cayman Chemical), RTG (Cayman Chemical), methotrexate (MTX, Sigma-Aldrich), and trans-ISRIB (ISRIB, Cayman Chemical) were resuspended in sterile DMSO (Sigma-Aldrich) at the following concentrations in aliquots for long- (−80°C) and short-term (−20°C) storage: ISRIB, 5mM; DTG, 10mM; RTG, 10mM; MTX, 20mM. Aliquots were thawed, and drugs were added to forebrain induction and differentiation media to achieve the following working concentrations: DTG: 0.1 μM, 1 μM, 10 μM; RTG: 10 μM; MTX: 1 μM; ISRIB: 5 μM. ISRIB was delivered together with 10.0 μM DTG for the duration of the treatment. During the drug treatment period (DIV 7−DIV 19 or DIV 7−DIV 39), media with the various drug concentrations was changed every day and organoids were collected on DIV 20 or DIV 40 for tissue processing. The selection of drug concentrations for DTG and RTG was informed by literature reporting plasma peak concentration values (C_max_) of approximately 8 μM for DTG and 3.4 μM for RTG in adults (Cottrell et al., 2013). Pharmacokinetic profiling during pregnancy has shown a decrease in C_max_ values in maternal plasma during the second and third trimesters, although the cord blood/maternal plasma concentration ratio was 1.25, suggesting high placental transfer (Mulligan et al., 2018). Our highest dose of DTG slightly exceeds the reported C_max_ value but falls within the range of cord blood concentration values reported in the literature (Figure 1A). Our dose of RTG is higher than the reported C_max_ value but matches the highest dose of DTG at 10 μM. The dose of methotrexate was based on literature showing neurotoxicity at 1 μM (Bruce-Gregorios et al., 1991).

### 2.4 Tissue processing and immunohistology

At experimental endpoints, brain organoids were placed directly in 16% methanol-free formaldehyde (Polysciences) diluted in DPBS (Gibco) overnight at 4°C on a rocking shaker (see Supplementary Table 1 for catalog numbers for all reagents). After fixation, brain organoids were washed in DPBS and cryoprotected by overnight incubation in 30% sucrose (Sigma-Aldrich) in DPBS at 4°C. Brain organoids were placed in a plastic cryomold (Electron Microscopy Sciences) and snap frozen in tissue freezing medium (General Data) on dry ice. Frozen tissue was stored at −80°C until processing. Serial tissue sections (25 μm for brain organoids) were sliced using a cryostat (Leica, CM3050S), and melted onto charged slides (Fisher Scientific). Slides were dried at room temperature and stored at −20°C until ready for immunohistological processing. For immunofluorescence staining, the tissue sections were outlined with a hydrophobic pen (Vector Laboratories) and washed with Tris-buffered saline containing 0.05% Tween-20 (TBST, Sigma-Aldrich). Tissue sections were permeabilized and non-specific binding was blocked using a solution containing 10% donkey serum (v/v), 0.5% Triton X-100 (v/v), 1% Bovine Serum Albumin (w/v), 0.1% gelatin (w/v), and 22.52 mg/ml glycine in TBST for 1 h at room temperature. The tissue sections were incubated with primary antibodies diluted in TBST with 5% donkey serum (Jackson ImmunoResearch) and 0.1% Triton X-100 (v/v) overnight at 4°C. We selected primary antibodies to observe organoid structure (laminin, PKCλ, Nestin), post-mitotic neurons (TBR1, NeuN), stem cells (SOX2), and cell cycle regulation (P21, CDT1), which were incubated at the following concentrations: CDT1 (Cell Signaling, 8064; 1:200), Laminin (Sigma-Aldrich, L9393; 1:500), Nestin (Abcam, ab105389; 1:300 and Santa Cruz, sc-21247; 1:500), NeuN (Sigma-Aldrich, ABN78; 1:500), P21 (Cell Signaling, 2946 and 2947; 1:400 and 1:500), PKCλ (BD Biosciences, 610207; 1:200), SOX2 (R& D Systems, AF2018; 1:500), TBR1 (Abcam, ab31940; 1:500). After washing in TBST, tissue sections were incubated with secondary antibodies (see Supplementary Table 1) diluted in TBST with 5% donkey serum (v/v) and 0.1% Triton X-100 (v/v) for 1.5 h at room temperature. After washing with TBST, sections were washed with TBS to remove detergent. After washing with TBS, slides were mounted in mounting solution (Vector Laboratories) and cover slipped.

### 2.5 Confocal microscopy and image processing

Brain organoid sections were imaged as z stacks using a Zeiss LSM 810 confocal microscope or a Zeiss LSM 710 confocal microscope (Zeiss) using a 10X, 20X, or 40X objective with Zen 2 software (Zeiss). Images were analyzed using ImageJ and RStudio software. For clearer visualization of organoid structures in the representative images used in the figure illustrations, composite images of Z stack projections were further processed with sequential functions in ImageJ: Enhance Local Contrast [CLAHE (blocksize: 127; histogram bins: 256; maximum slope: 3.00)]; Subtract Background (sliding paraboloid with a radius of 50 pixels) and/or Noise-Despeckle. All functions were applied to the entire composite image and not to specific regions or channels. These additional enhancements to the image were not applied prior to quantification.

### 2.6 RNA extraction and RNA-sequencing

To minimize organoid-to-organoid variability, 5–10 organoids were pooled into one sample for analysis. Organoids were homogenized in 400 μL TRIzol (Invitrogen) by manual pipetting until complete lysis (see Supplementary Table 1 for catalog numbers for all reagents). RNA was isolated by the addition of 80 μL chloroform (Sigma-Aldrich), vortexing and centrifugation at 4°C for 15 min at 12,000 g, and careful collection of 200 μL of the upper aqueous layer. RNA clean-up was performed using a silica spin column (Zymo) according to the manufacturer’s instructions, followed by elution in 15 μL water and analysis by NanoDrop 2000 (Thermo Scientific). RNA was immediately extracted, minimizing any additional steps that could lead to RNA degradation, which preserves RNA integrity for further processing. RNA-seq library preparation was performed as previously described (Jacob et al., 2020; Weng et al., 2018). Sequencing was performed on a NextSeq 550 (Illumina) with 3.0 pM loading, using 1x75bp reads. Raw sequencing data was demultiplexed with bcl2fastq2 v.2.19.0.316 (Illumina) with adapter trimming turned off. Alignment was performed using STAR v.2.6.1d (Dobin et al., 2013) with GRCh38 used as the reference genome and gencode v.28 GTF used as the annotation file. Multimapping and chimeric alignments were discarded, and uniquely mapping reads were quantified at the exon level and summarized to gene counts using python package HTSeq v0.11.1 (Anders et al., 2015). Differential gene expression analysis was performed using the DESeq2 suite of analysis tools (Love et al., 2014). Gene ontology analysis was performed using the Database for Annotation, Visualization and Integrated Discovery (DAVID) (Sherman et al., 2022).

### 2.7 Statistical analysis

All statistical tests and sample sizes are included in the figure legends and text. All data are shown as means ± SEM as stated in the figure legends. Statistical analyses and data visualization were performed using GraphPad PrismV10. For all quantifications of immunohistology, the samples being compared were cultured and processed in parallel and imaged using the same settings and laser power for confocal microscopy. Two- or one-way ANOVA was performed as indicated in the figure legends with Dunnett’s tests for multiple comparisons.

Immuno-positive cells were identified using automated quantification generated using ImageJ macros and RStudio scripts. Briefly, original CZI confocal images were orthogonally projected (Zen) and converted into TIFF file format (ImageJ) to separate the images’ channels. Images of each channel from each immunostaining condition were processed together (ImageJ) to calculate fluorescence intensity of each nucleus identified in the image by the software based on the DAPI channel. Mean intensity values for each cell nucleus in each channel were organized into CSV files (Microsoft) which were then imported into RStudio for automated cell counting based on each image’s threshold to account for variance in image quality. Image thresholds for marker positivity were determined using the automatic threshold function in ImageJ. Immuno-positive cell counts and ratios (relative to SOX2^+^, DAPI^+^, or other antibodies of interest) were organized into CSV files (Microsoft) and imported into GraphPad Prism for analysis and visualization. Organoid circumference was measured using images from organoid sections subjected to immunostaining to identify rosettes (SOX2, LAMININ, PKCλ). All images were measured using the ImageJ freehand drawing or ellipse tool to outline the organoid and the automated measure tool to calculate the perimeter based on the calibrated scale for image magnification at 20x. Rosettes were identified with PKCλ staining at the ventricular surface and radial orientation of SOX2^+^ cells. All rosettes were counted in each organoid imaged at 20X. All images were blinded during analysis.

## 3 Results

### 3.1 DTG exposure leads to reduced size of cortical organoids

We first sought to determine whether chronic exposure to DTG would impact the overall size and growth of our forebrain cortical organoids. The drug concentrations for DTG were selected based on literature reporting umbilical cord blood concentrations, as a proxy for fetal exposure, ranging between approximately 3 to 20 μM (Lewis et al., 2016; Mulligan et al., 2018; Pain et al., 2015; Rimawi et al., 2017; Schalkwijk et al., 2016; Figure 1A). We used several concentrations of DTG (0.1, 1.0, and 10.0 μM), 10.0 μM of RTG, another antiretroviral INSTI (Steigbigel et al., 2008), and 1.0 μM of methotrexate (MTX), a drug with known teratogenicity (Hyoun et al., 2012). Organoids were exposed to the drugs or the vehicle control (DMSO) through complete daily media changes and drug or vehicle replacement starting at DIV 7 and collected at DIV 20 or DIV 40, the two endpoints for analysis (Figure 1B). These stages are transcriptionally similar to the developing fetal cortex at approximately 8 weeks post-conception (Day 20) or 12–24 weeks post-conception (Day 40) (Amiri et al., 2018; Camp et al., 2015; Qian et al., 2016). Since DTG exposure at the time of conception was associated with a slight increase in the risk for neural tube defects in the initial report from Botswana (Zash et al., 2018), we focused most of our analyses on Day 20 organoids to capture the earliest stages of development in our organoid model.

To evaluate the structure of the organoids, we analyzed organoids from three iPSC lines at Day 20 following 12 days of exposure to DTG, using three differentiation batches for each line (n = 3 organoids per batch/per line). A two-way ANOVA was performed to evaluate the effects of cell line and drug treatment on organoid size as measured by the circumference of sections that were imaged following immunohistological processing. The results indicated significant main effects for cell line (F_8, 148_ = 8.636, *p* < 0.0001) and drug treatment (F_5, 148_ = 6.864, *p* < 0.0001). Post hoc analyses for drug treatment effects across all cell lines revealed a smaller size of organoids treated with 10 μM DTG (p = 0.0006) or 1 μM MTX (p < 0.0001) compared to organoids exposed to DMSO (Figures 1C, D).

Neural differentiation of embryonic stem cells or iPSCs can lead to the formation of rosette structures that consist of radially organized cells expressing early markers of neuroectoderm (Wilson and Stice, 2006; Zhang et al., 2001). These structures can form in 3D organoids and have broad neural differentiation potential, reminiscent of neural progenitors at the neural plate stage before neural tube closure (Elkabetz et al., 2008). We identified rosette structures based on a visible lumen structure, often labeled with the apical marker PKCλ, and radial organization of progenitors revealed by alignment of NESTIN^+^ fibers and/or alignment of SOX2^+^ cells (Figure 1C). We counted the total number of rosettes in sections of whole organoids imaged at 20X, divided by the approximated area, to obtain a measure of rosette density (Figure 1E). Two-way ANOVA revealed an effect of both cell line (F_8, 148_ = 20.44, *p* < 0.0001) and drug treatment (*F*_5, 148_ = 60.91, *p* < 0.0001). *Post-hoc* analyses revealed a significant effect of 10 μM DTG exposure, compared to DMSO exposed-organoids, on the density of rosettes detected. Different from the measurement of overall size, exposure to MTX did not have a significant effect on the number of rosettes when compared to organoids treated with DMSO. Finally, we performed Caspase 3 staining to evaluate cell death in each of the conditions using a single batch of each cell line (Supplementary Figure 1). There was some variability across lines and a two way ANOVA showed an effect for both cell line and treatment condition (cell line: *F*_2,32_ = 10.00, *p* = 0.0003; drug treatment: *F*_4,42_ = 3.494, *p* = 0.0150). *Post-hoc* analysis showed a significant effect of an increase in Caspase 3 staining among Sox2^+^ cells for the 10 μM DTG condition compared to DMSO, but this was likely driven by a large increase in Caspase 3 expression in one cell line. Notably, exposure to RTG had no appreciable effect on the size of the organoids or the number of rosettes compared to DMSO-exposed organoids, demonstrating that the impact from DTG on organoid structure was not generalized to all INSTIs.

### 3.2 DTG induces gene expression changes related to neurogenesis and cellular stress

**FIGURE 2:**
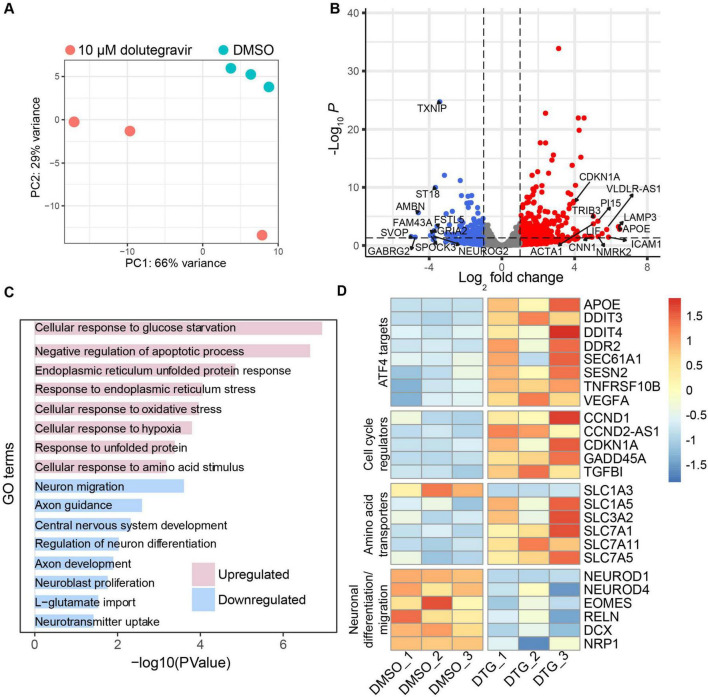
RNA-seq reveals upregulation of stress-related genes and downregulation of neurogenesis-related genes. **(A)** Principal components analysis (PCA) plot of bulk RNA-seq data from organoids treated with DMSO or 10.0 μM DTG for 12 days and analyzed at Day 20 (C1-2 cell line, three replicates for each condition) showing variability within and across conditions. The plot was generated using the “plotPCA” function embedded in the DESeq2 package. **(B)** Volcano plot showing downregulated (blue) and upregulated (red) genes. Differential gene expression analysis based on a model using negative binomial distribution, DESeq2 with Benjamini-Hochberg correction. The top 10 downregulated and upregulated genes, as well as CDKN1A, are labeled. **(C)** Functional enrichment analysis showing select upregulated and downregulated gene sets using DAVID (Sherman et al., 2022). **(D)** Heat map, generated using the R package “pheatmap”, showing expression levels of genes of interest based on functional properties including gene targets of ATF4, which is an effector of the integrated stress response, cell cycle regulators, amino acid transporters, and genes related to neurodevelopment and migration.

To investigate the cellular processes that could be driving the reduction in size and rosette formation in the organoids, we performed bulk RNA-seq in the C1-2 iPSC line to identify differentially expressed genes following exposure to the highest dose of DTG. We collected organoids from 3 batches for analysis at DIV 20 and observed minimal variation in DMSO-treated organoids, which were distinct from organoids treated with 10 μM DTG from DIV 7 through DIV 19 (Figure 2A). Among the 373 differentially expressed genes (DEGs) in DTG-treated organoids compared to DMSO-exposed controls with significant differences in expression, we observed 256 upregulated and 117 downregulated genes (Figure 2B and Supplementary Table 2). Significant differentially expressed genes were determined based on a fold-change of at least 2 and a p-value of less than 0.05 after adjustment for multiple testing. Gene ontology (GO) analysis via DAVID (Sherman et al., 2022) revealed upregulation of gene sets related to cellular stress pathways and cellular response to glucose starvation and amino acid stimulus (Figure 2C and Supplementary Table 3). GO terms extracted from the list of downregulated genes implicated processes related to neuron differentiation and migration, central nervous system development, and neuroblast proliferation (Figure 2C and Supplementary Table 3).

To further investigate the nature of the cellular stress response, we initially evaluated components of the ISR based on previous studies that have reported ISR activation in neurons following exposure to some antiretroviral drugs, such as elvitegravir (Stern et al., 2018). The ISR is induced through the activation of one of four kinases, leading to phosphorylation of the eukaryotic translation initiation factor 2 (eIF2α), which in turn inhibits the activity of eIF2B leading to a global reduction in mRNA translation (Costa-Mattioli and Walter, 2020). However, selective transcripts with a short inhibitory upstream open reading frame (uORF), such as activating transcription factor 4 (ATF4), are preferentially translated (Vattem and Wek, 2004). We identified several ATF4 target genes that were upregulated in our DTG-exposed organoids (Figure 2D). Among these were DNA damage-inducible transcript 3 (DDIT3), also known as CHOP, and tumor necrosis factor receptor superfamily member 10B (TSFRSF10B), also known as DR5, which have been implicated in apoptotic pathways triggered by the unfolded protein response in the endoplasmic reticulum (ER) (Lam et al., 2020). Upregulation of another ATF4 target, Sestrin-2 (SESN2), further suggests the potential involvement of oxidative stress, ER stress (Lu et al., 2023), and the regulation of mTORC1 activation (Wolfson et al., 2016).

Similar to ATF4, cyclin dependent kinase inhibitor 1A (CDKN1A) has a uORF that allows translation during activation of the ISR and was one of several cell cycle regulatory genes upregulated in the organoids exposed to DTG compared to vehicle-treated organoids (Kanehisa et al., 2023; Figure 2D). CDKN1A and GADD45α are regulators that can lead to arrest at the G_1_/S and G_2_/M cell cycle checkpoints, respectively, and have been shown to interact with each other (Kearsey et al., 1995; Lehman et al., 2015; Wang et al., 1999). Although expression and function of transforming growth factor β1 (TGF- β1) signaling is context-dependent, it can also lead to G_1_ arrest and may contribute to a positive feedback loop through induction of CDKN1A to further promote G_1_/S cell cycle arrest (Datto et al., 1995; Misumi et al., 2008).

Interestingly, several members of the amino acid transporter Solute Carrier protein (SLC) superfamily were upregulated in organoids following DTG exposure (Figure 2D). Members of the SLC1 family are primarily involved in glutamate transport and maintaining low levels of extracellular glutamate, while SLC3/7 family members, such as SLC3A2 and SLC7A5, can form heterodimers that are critical for the transport of most essential amino acids (Pizzagalli et al., 2021). SLC family genes, several of which were upregulated in organoids exposed to DTG, have also been shown to mediate amino acid activation of the mTORC1 pathway (Nicklin et al., 2009), which has broad effects on neurogenic processes and cortical development (Nguyen et al., 2024).

Downregulated genes related to neural development included members of the neural lineage basic-Helix-loop-Helix transcription factor family such as NeuroD1, which has been shown to mediate neurogenesis, epigenetic programming of fate specification, and neuronal migration (Pataskar et al., 2016; Tutukova et al., 2021), and NeuroD4 (Math3), a driver of cortical gene expression in the dorsal telencephalic progenitors to promote glutamatergic neuron identity (Mattar et al., 2008). Neurogenesis is also likely affected by the downregulation of the T-box gene Tbr2/EOMES at this early stage of organoid development, as it is highly expressed in intermediate cortical progenitors in mice (Mihalas and Hevner, 2017) and humans (Cipriani et al., 2016) as part of a transcriptional cascade during glutamatergic neurogenesis. Downregulation of RELN at this stage would likely impact laminar organization and neuronal migration as it is typically highly expressed during the expansion of the cortical plate during cortical neurogenesis (Meyer, 2010). Together these up- and downregulated genes in DTG-exposed organoids are consistent with a cumulative effect on human forebrain organoid development, including dysregulation of amino acid signaling in neural progenitors, leading to the induction of cellular stress responses and a disruption of transcriptional programs that support cortical neurogenesis.

### 3.3 Reduced neurogenesis in DTG-exposed organoids

**FIGURE 3:**
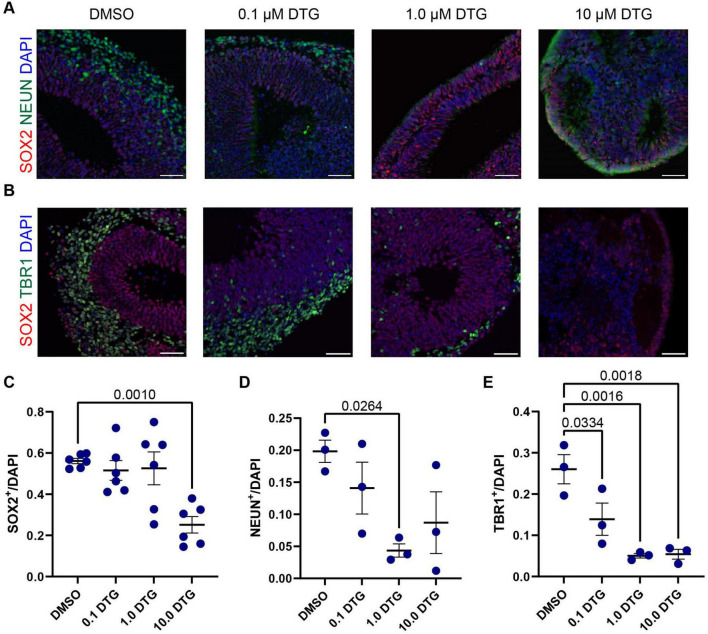
Exposure to DTG reduces the number of postmitotic neurons in organoids. **(A,B)** Representative images of selected regions in Day 40 organoids from the C1-2 iPSC line following daily exposure to DTG from Day 7 to Day 39. Scale bars, 100 μm. **(A)** Expression of NEUN, a marker expressed in most postmitotic cortical neurons. **(B)** Expression of TBR1, a transcription factor expressed in postmitotic neurons during glutamatergic neuron development and SOX2, a marker of neural stem cells. Quantifications show a reduction in the proportion of SOX2^+^ neural stem cells **(C)**, and postmitotic neurons expressing NEUN **(D)** or TBR1 **(E)** among DAPI cells under some conditions. Data points represent marker expression quantified from a single organoid region, with *n* = 3 organoid regions for TBR1 and NEUN expression and *n* = 6 for SOX2 expression. One-way ANOVA with Dunnett’s test for multiple comparisons, adjusted p values shown for statistically significant differences among pairwise comparisons. Data shown are means ± SEM.

Based on the observation of neurogenesis-related genes that were downregulated following DTG exposure at Day 20 (Figures 2C, D) and the overall reduction in size in DTG-treated organoids, we next quantified the number of neural stem cells and postmitotic neurons in DIV 40 organoids generated from the C1-2 cell line that had been exposed to DTG starting on DIV 7 (Figures 3A, B). For quantification, we selected a region of the organoid with the most prominent rosette structure to identify neural stem cells, marked with SOX2, which are typically located proximal to a ventricle-like area, and postmitotic neurons, marked with NEUN or TBR1, which are typically located at the perimeter of the rosette. We normalized the expression of SOX2, TBR1, and NEUN to DAPI to account for differences in the areas of quantification. We first observed a decrease in the percentage of neural stem cells marked by SOX2 expression in organoids treated with the highest dose of DTG (Figure 3C). We next quantified the percentage of cells expressing NEUN, a commonly used marker of postmitotic neurons that is expressed in most neocortical populations (Sarnat et al., 1998). We observed a reduction in the number of NEUN^+^ neurons in organoids treated with 1.0 μM DTG and a trend towards a reduction of NEUN^+^ neurons in organoids treated with lower (0.1 μM) and higher (10.0 μM) doses of DTG that was variable and not significantly different from DMSO-treated organoids (Figure 3D). Finally, we counted the number of cells expressing TBR1, a member of the T-box family of transcription factors. TBR1 is expressed in early-born postmitotic neurons of deep cortical layers and has also been implicated in the regulation of neuronal migration and the laminar organization of the cortex (Bedogni et al., 2010). We observed a decrease in the number of TBR1^+^ cells at all doses of DTG-treated organoids compared to DMSO-exposed organoids (Figure 3E). Together, these data suggest that neurogenesis is impaired in organoids treated with DTG, with fewer neurons and a smaller pool of neural stem cells.

### 3.4 Downstream effectors of the integrated stress response are upregulated following chronic exposure to DTG

**FIGURE 4:**
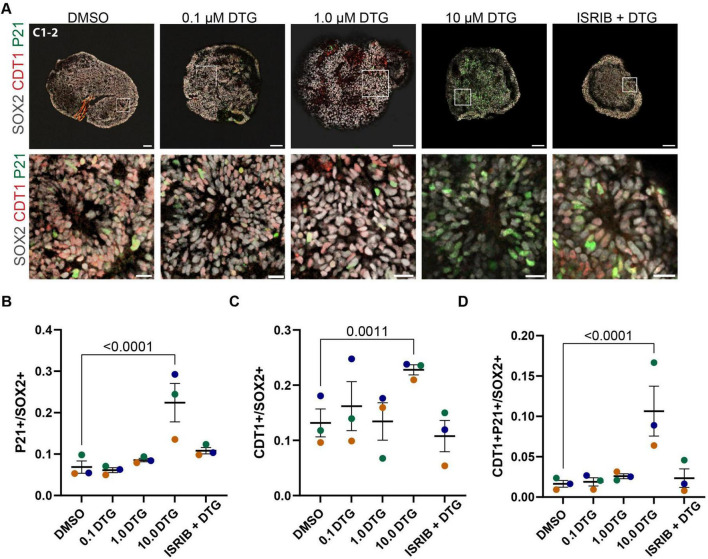
Upregulation of a cell cycle regulator and effector of the integrated stress response. **(A)** Representative images from the C1-2 line treated with DMSO, 0.1, 1.0, 10.0 μM DTG, or 10.0 μM DTG together with 5 μM ISRIB, an inhibitor of the integrated stress response (top row, scale bars = 100 μm) with magnified view of regions demarcated by white boxes (bottom row, scale bars = 20 μm). **(B–D)** Quantification of the proportion of P21^+^ **(B)**, CDT1^+^ **(C)**, and double-positive P21^+^CDT1^+^ cells among SOX2^+^ marked cells **(D)**. Data points represent marker expression averaged over 3 organoids per line. Marker color represents cell line identity. Main effects of both cell line and drug treatment for CDT1 expression (cell line: *F*_2,38_ = 7.251, *p* = 0.0022; drug treatment: *F*_4,30_ = 10.65, *p* = 0.0002). Main effect of drug treatment for P21^+^ (*F*_4,38_ = 14.78, *p* < 0.0001) and P21^+^ CDT1^+^ (*F*_4,38_ = 9.244, *p* < 0.0001) among SOX2^+^ cells. Post hoc analyses with Dunnett’s tests for multiple comparisons revealed a higher percentage of P21^+^, CDT1^+^, and P21^+^ CDT1^+^ among SOX2^+^ cells with exposure to the highest dose of DTG compared to DMSO-exposed organoids. Adjusted *p*-values are shown for statistically significant differences among pairwise comparisons. Data shown are means ± SEM.

Given the upregulation of ATF4 and its target genes detected in the RNA-seq analysis and previous reports showing that some ART drugs lead to activation of the ISR in cultured rat neurons (Stern et al., 2018), we next considered whether any downstream effectors of the ISR could play a role in the deficits we observed. In addition to ATF4, activation of the ISR leads to enhanced translation of variant 4 of CDKN1A, which encodes the protein P21 (Lehman et al., 2015). P21 has been shown to inhibit cyclin E:CDK2 complexes, leading to arrest of the cell cycle in G_1_/S phase (Shackelford et al., 1999). We examined P21 expression in the population of SOX2-expressing neural stem cells (Figure 4A). Two-way ANOVA revealed an effect of drug treatment (*F*_4,38_ = 14.78, *p* < 0.0001) and post hoc analysis showed that the percentage of cells expressing P21 was significantly higher in organoids treated with the highest dose of DTG compared to organoids exposed to DMSO (*p* < 0.0001) (Figure 4B). P21 can be upregulated in response to ISR activation and therefore we investigated whether the small molecule ISR inhibitor, ISRIB, could rescue any of the effects of DTG. Co-application of the highest dose of DTG (10 μM) with ISRIB (5 μM) did ameliorate the increase in P21 expression as levels were comparable to DMSO-exposed control organoids (*p* < 0.2843) (Figure 4B). Since P21 is implicated in cell cycle arrest and, in particular, the G_1_/S transition, we next probed expression of CDT1, which accumulates in the G_1_ phase and is degraded at the onset of the S phase (Tada, 2007). We observed an increase in CDT1 expression in 10 μM DTG-treated organoids, but not at lower concentrations, and this effect was rescued in the organoids concurrently treated with ISRIB (Figure 4C). Two-way ANOVA showed significant main effects for cell line (*F*_2,38_ = 7.251, *p* = 0.0022) and drug treatment (*F*_4,38_ = 7.324, *p* = 0.0002) and a post hoc test showed a significant effect for the highest dose of DTG (p = 0.0011) but not for DTG with ISRIB (*p* = 0.7201) when compared to DMSO-treated organoids. Finally, we examined co-expression of CDT1 and P21 among SOX2^+^ cells and observed a higher percentage of CDT1^+^P21^+^ cells among SOX2^+^ cells following exposure to the highest dose of DTG compared to vehicle-treated organoids, which was not observed with lower doses of DTG or in organoids co-treated with the highest dose of DTG and ISRIB together (Figure 4D). Two-way ANOVA showed a significant impact of drug treatment (F_4,38_ = 9.244, *p* < 0.0001) with post hoc comparisons showing a significant effect only for 10 μM DTG (*p* < 0.0001) compared to vehicle-treated organoids. These data further support that DTG exposure leads to activation of the ISR and upregulation of a downstream effector, P21, which could disrupt cell cycle progression and impair neurogenesis.

## 4 Discussion

Here, we show that forebrain cortical organoids exposed to a high dose of DTG have a reduced size and number of neural rosettes compared to vehicle-treated organoids, as well as signatures of transcriptional dysregulation suggesting the induction of stress response pathways to impair neurogenesis. Consistent with activation of the ISR, upregulation of P21, an effector of cell cycle arrest, was observed in organoids treated with the highest dose of DTG, which was ameliorated by the co-application of an ISR inhibitor, ISRIB. Exposure to RTG, another INSTI, had a minimal impact on organoid growth and rosette formation, suggesting the structural deficits we observed were not broadly induced by all ART drugs in the same mechanistic class. Together, these data support the use of human iPSC-based models to screen for deleterious effects of drugs in human neural cell types that may inform the evaluation of current therapeutic approaches and the development of new drugs.

Many therapeutic drugs are clinically tested in a population that specifically excludes pregnant people. Data regarding safety for the use of many drugs in pregnant people are often based on observational studies performed after drug approval (Ren et al., 2021). One such surveillance study was conducted in Botswana as part of the Tsepamo Study, which spanned the transition from efavirenz to DTG as a first-line treatment for PLWH (Republic of Botswana, 2016). DTG is a generally well-tolerated FDA-approved ART drug that inhibits HIV-1 transmission and viral production through blocking integrase and preventing the integration of viral DNA. Following the adoption of DTG as a frontline ART drug in Botswana, reports from an observational study suggested that there may be a slight increase in the risk for neural tube defects following DTG exposure at the time of conception (Zash et al., 2019; Zash et al., 2018), which led several national and international health agencies to issue statements of concern. More recent reports have suggested the risk of neural tube defects is minimal or not detected following periconceptual DTG exposure (Kourtis et al., 2023; Patel et al., 2022; Pereira et al., 2021), and currently, both the U.S. Department of Health and Human Services and the World Health Organization list DTG as an approved ART drug for use during pregnancy or by people trying to conceive (United States Department of Health and Human Services, 2024; WHO, 2021). However, the global impact of changing treatment regimen recommendations within a short period of time and the need to wait until more information is available from surveillance studies highlight the necessity for alternative sources of safety data. In addition, although the risk of adverse birth outcomes and occurrence of major anomalies in utero due to DTG exposure appears to be minimal, there may be more subtle effects on fetal development that would not be as easily detected in these observational studies. Further, some of the metabolic effects of DTG may impact maternal health and indirectly affect perinatal neurodevelopment (Dontsova et al., 2023). Although data are currently limited, there are questions about the long-term or delayed consequences of developmental exposure to DTG, which have been the subject of investigation in animal models and ongoing observational studies (Foster et al., 2022).

As ART has become increasingly affordable and accessible to the childbearing population across the globe, the number of HIV-exposed but uninfected children is on the rise, almost all of whom were exposed to ART perinatally (Evans et al., 2016). Recent studies have begun to investigate whether children with sustained perinatal exposure to ART are subject to developmental delays or neurological impairments (Caniglia et al., 2018; Whitehead et al., 2014), and results have been equivocal, with some studies showing minimal effects of ART drugs on any gross measure in early development (Coelho et al., 2017; Spaulding et al., 2016) and other studies suggesting the presence of congenital abnormalities (Williams et al., 2015) or behavioral and cognitive deficits that appear later in development (Smith et al., 2017). A recent study in Kenya found that several factors, including in utero exposure to efavirenz, but not DTG, were associated with lower neurodevelopmental scores in 1-year-old children exposed to HIV (Bulterys et al., 2023). Relevant to postnatal development, RTG and DTG are the currently preferred INSTIs for neonates and infants ≥ 4 weeks of age living with HIV, respectively (WHO, 2021), and several studies have assessed the safety and efficacy of both RTG and DTG in children and adolescents, with a systematic review suggesting there are no safety concerns about treatment in this population (Townsend et al., 2022).

Using our forebrain organoid platform as a model of early cortical development, we focused on the direct effects of DTG on human neural progenitor cells and identified structural effects of DTG on forebrain organoids that did not extend to RTG, another INSTI (Figure 1). DTG reduced overall growth and decreased the number of neural rosettes that were formed in organoids treated with the highest dose of DTG compared to organoids exposed to the vehicle alone. There were no apparent differences in organoids treated with the same dose of RTG (10 μM) compared to vehicle-treated organoids, demonstrating that the structural deficits we observed following DTG exposure were not due to general toxicity or mechanisms common to all INSTIs (Supplementary Figure 1). Importantly, our drug dose (10 μM) was a slightly higher concentration than the peak plasma concentration reported for DTG (∼8 μM) (Cottrell et al., 2013) but more than three times higher than the reported peak concentration for RTG (∼3 μM) (Ene et al., 2011). Although it is difficult to correlate plasma concentrations with cell culture conditions, these data suggest that the discrepancy between the ART drug-treated organoids is not due to a selective supraphysiological dose of DTG rather than RTG. To further validate the model, we exposed organoids to MTX, a folic acid antagonist that is used clinically to treat cancer and rheumatoid arthritis but has well-established teratogenic effects, including neural tube defects (Lloyd et al., 1999). Similar to DTG, MTX exposure resulted in a reduction in the overall size of organoids compared to vehicle-treated organoids. However, the relative number of rosettes did not differ between vehicle- and MTX-treated organoids, suggesting a dissociation between these phenotypes in our model in response to two drugs that impacted overall organoid growth.

One factor that may have contributed to the early signal of elevated risk of neural tube defects in the observational study is the lack of a public folic acid fortification program in Botswana. Recent studies have supported a potential interaction between DTG and folate metabolism, finding that DTG is a partial antagonist of folate receptor 1 and impacts the expression of folate transporters (Cabrera et al., 2019; Gilmore et al., 2022). Studies in mice have also shown an interaction between low folic acid intake and DTG exposure leading to neural tube defects (Tukeman et al., 2024). One study reported that maternal folate deficiency led to a downregulation of amino acid transporters, which inhibited the mTORC1 pathway and impaired fetal growth in mice (Rosario et al., 2017). mTORC1 is a sensor for amino acid availability and a master regulator of cell growth (Nicklin et al., 2009). mTORC1 has also been shown to increase expression of ATF4, which is a major effector of the ISR that can be initiated by dysregulated amino acid sensing through GCN2 to phosphorylate eIF2α. Interestingly, gene targets of mTORC1-dependent activation of ATF4 are a subset of those upregulated in response to ISR-dependent activation (Torrence et al., 2021). Among the upregulated ATF4 targets we detected in our RNA-seq data (Figure 2D), several are common to both mTORC1- and ISR-dependent ATF4 signaling, including SESN2, DDIT3, DDIT4, and the amino acid transporters, SLC3A2 and SLC7A5 (LAT1). ATF4-mediated upregulation of SESN2 and DDIT4 inhibits mTORC1 activity in response to sustained deprivation of amino acids such as leucine, serving as a negative feedback signal to suppress mTORC1 signaling and downregulate amino acid transporters (Xu et al., 2020). Our data are consistent with ATF4 acting as a convergent sensor of amino acid availability through both mTORC1 and GCN2/peIF2α signaling (Park et al., 2017), while further suggesting that the negative feedback from SESN2 and DDIT4 are insufficient to downregulate the expression of amino acid transporters. It remains to be determined whether there is sustained dysregulation of amino acid transporters in this model and how the dynamics of mTORC1 and ISR activation may contribute to cellular phenotypes under conditions of chronic exposure to DTG.

The ISR is an adaptive program in response to acute extrinsic or intrinsic stress that broadly suppresses translation as a protective measure until cellular homeostasis is restored. However, prolonged or severe stress can lead to the sustained activation of the ISR and the initiation of processes leading to cell death, due in part to downstream effectors such as ATF4 and its targets, such as DDIT3 (CHOP) (Pakos-Zebrucka et al., 2016). In addition to ATF4, the ISR also leads to an increase in the translation of one variant of CDKN1A, which encodes the cell cycle inhibitor P21. Upregulation of P21 can lead to cell cycle arrest at the G_1_/S transition (Lehman et al., 2015). We observed increased expression of both P21 and CDT1, which accumulates in the G_1_ phase of the cell cycle, in neural stem cells in organoids treated with the highest dose of DTG compared to vehicle-exposed organoids (Figure 4). These data provide additional support for the involvement of the ISR in our model and a potential mechanism underlying the impairment in neurogenesis and the lower number of neurons observed in organoids exposed to DTG for 32 days (Figure 3), compared to vehicle-treated organoids. Taken together, our data support a role for the ISR in DTG-mediated cellular phenotypes and a plausible contribution of GCN2-mediated phosphorylation of eIF2α, leading to post-transcriptional regulation of ATF4 and CDKN1A. Future studies are needed to determine whether GCN2 is responsible for ISR activation and if so, whether this is a cause of the cellular response to DTG, or a consequence of another primary insult that dysregulates amino acid homeostasis.

Historically, concerns about legal liability, potential harm to the fetus, and pregnancy-induced changes in physiology have limited the inclusion of pregnant people in clinical trials for drug development and testing, although recent revisions to the Common Rule and a growing recognition of the need to obtain these data may lead to systemic changes in the near future (Ren et al., 2021). At the present time, there are often minimal data on the safety and efficacy of recently developed therapeutic drugs. Indeed, for several ART drugs, including some of the recently developed formulations such as cabotegravir, the U.S. government website reports there are not sufficient data to evaluate teratogenicity in humans (United States Department of Health and Human Services, 2024), leaving the pregnant population and physicians with little data to guide clinical decisions when one or more drugs are approved in a therapeutic class. To bridge the gap between preclinical models and observational studies of pregnancy outcomes following drug exposure, human iPSCs provide a renewable resource to generate cell types of interest, including neural cells, for systematic investigation. Using iPSCs from multiple donors, we were able to show a selective impact of DTG, but not RTG, on neural stem cells and neurogenesis in a cortical organoid model. Together, these results illustrate the potential for iPSC-based strategies to reveal biological processes that may be affected by drugs during neural development and provide complementary data in relevant human cell types to augment preclinical investigations of drug safety. It is, however, important to note the limitations of in vitro models, which lack many properties of intact physiological systems that are particularly relevant for drug studies, including mechanisms for drug metabolism and clearance. These models are likely best suited as a means of rapid drug screening for toxicity and to investigate mechanisms of action and off-target effects that can inform the development of clinical studies, which provide definitive data on safety and efficacy to guide treatment decisions.

## Data Availability

The RNA-seq datasets generated for this study are deposited in the Gene Expression Omnibus (GEO) public repository (GSE275931) (https://www.ncbi.nlm.nih.gov/geo/query/acc.cgi?acc=GSE275931).
