# Supplementary material for: Exposure to the antiretroviral drug dolutegravir impairs structure and neurogenesis in a forebrain organoid model of human embryonic cortical development
Source: Front Mol Neurosci. 2024 Nov 6;17:1459877. doi: 10.3389/fnmol.2024.1459877 (PMC11576471; doi:10.3389/fnmol.2024.1459877)
Supplement: Supplementary file 4 [file Data_Sheet_1.docx]

**Supplementary Information**

**
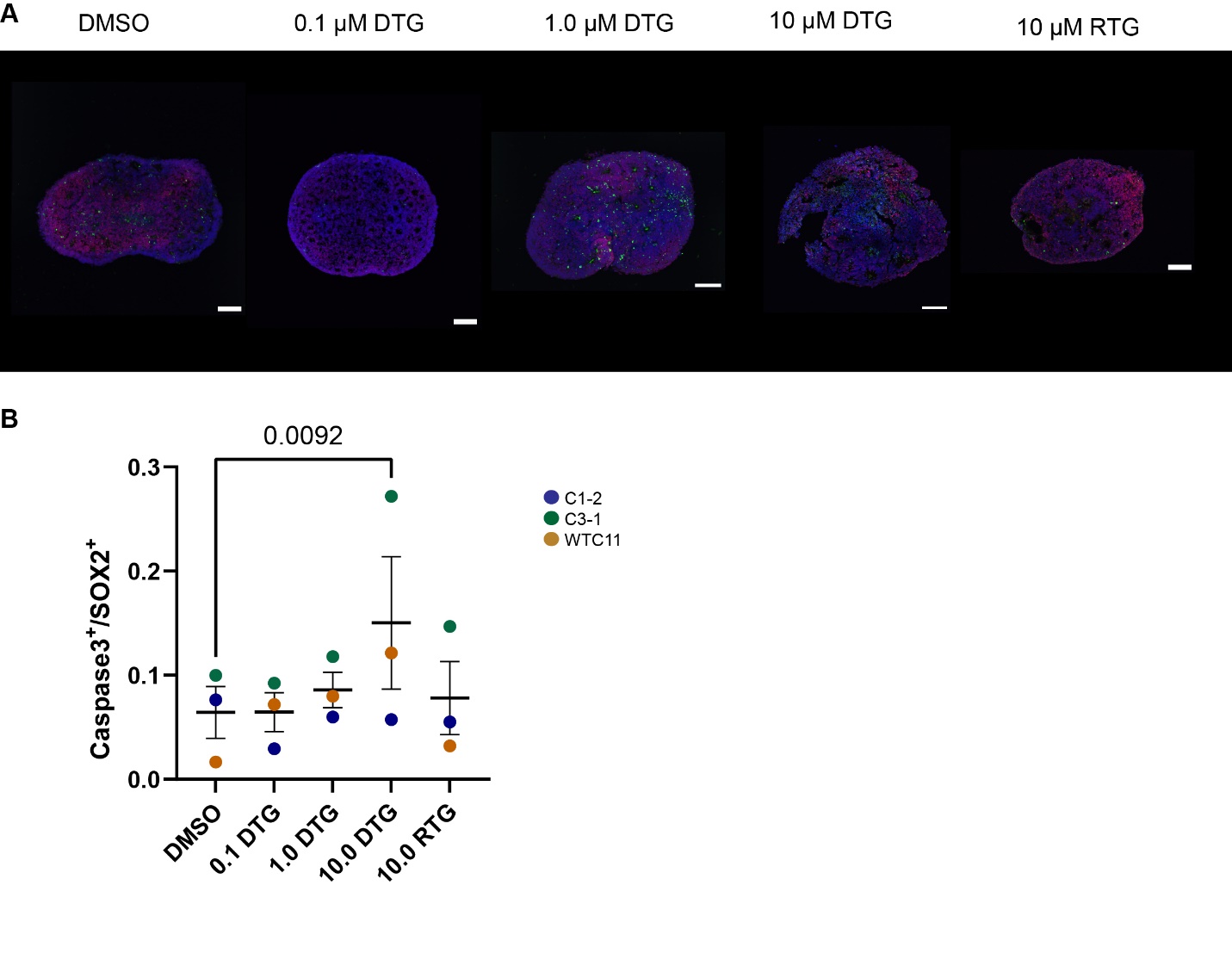
 Supplementary Figure 1. Caspase 3 expression.**

(A) Representative images from the WTC-11 line treated with 0.01% DMSO, 0.1, 1.0, or 10.0 µM DTG, or 10.0 µM RTG (scale bars = 100µm). (B) Quantification of Caspase 3^+^ (CASP3) cells among SOX2^+^ marked cells. Data points represent marker expression averaged over at least 3 organoids per line per treatment. Marker color represents cell line identity. Main effects of both cell line and drug treatment for CASP3 expression (cell line: F_2,32_ = 10.00, p = 0.0003; drug treatment: F_4,42_ = 3.494, p = 0.0150). Post hoc analyses with Dunnett’s tests for multiple comparisons revealed a higher percentage of CASP3^+^ among SOX2^+^ cells with exposure to the highest dose of DTG compared to DMSO-exposed organoids. Adjusted p value is shown for the statistically significant difference among pairwise comparisons. Data shown are means ± SEM.

**Supplementary Tables (In Excel files)**

**Supplementary Table 1. Key resources and reagents**

**Supplementary Table 2. List of differentially expressed genes (DEGs), related to Figure 2.**

**Supplementary Table 3. List of Gene Ontology terms in the analysis of upregulated and downregulated genes.**
